# Zoo and Aquarium Animal Welfare, Ethics, and Behavior

**DOI:** 10.3390/ani14111552

**Published:** 2024-05-24

**Authors:** David S. Miller, Kathleen M. Dudzinski, Raymond Anthony, Heather Manitzas Hill, Grey Stafford

**Affiliations:** 1 Miller Veterinary Services, PLLC, Loveland, CO 80539, USA; 2 Dolphin Communication Project, P.O. Box 7485, Port Saint Lucie, FL 34984, USA; kdudzinski@dolphincommunicationproject.org; 3 Department of Philosophy, University of Alaska Anchorage, Anchorage, AK 99508, USA; rxanthony@alaska.edu; 4 Department of Psychology, St. Mary’s University, 1 Camino Santa Maria, San Antonio, TX 78228, USA; hhill1@stmarytx.edu; 5 College of Science, Engineering and Technology, Grand Canyon University, 3300 West Camelback Rd., Phoenix, AZ 85017, USA; grey@ireinforce.com

This Special Issue of *Animals* was launched to promote the discussion of how animal welfare can be best addressed in zoos and aquariums while accommodating conservation needs, as well as how further improvements can be made in a similar vein to how medical and scientific research has advanced human health and welfare. The contributing authors illustrate the thoughtfulness that many professionals have invested in continually improving animal welfare in these settings. The Guest Co-Editors are sensitive to a diversity of philosophical views about the morality of keeping animals in captivity; they firmly believe that when human beings are responsible for the lives of animals, the needs and conditions of wildlife in zoos and aquariums can be met through transparently communicated and rigorously deliberated scientific research that is evaluated for its merits, rather than political or personal strategies that are divorced from the realities that animals experience. This is how animal science and welfare and the conscientious stewardship of animals have progressed over the centuries. It is the only path whereby zoo and aquarium animal welfare and conservation policy and management can be improved continually and avoid complacency. With this perspective, we thank *Animals* for supporting a range of papers that include the ethics and legal concerns of zoo and aquarium species within an environment of diverse societal values, strategies for improving animal welfare, and the need to critically evaluate the data surrounding controversial topics. A relevant example is distinguishing between philosophical beliefs and science-supported views of the welfare of marine mammals under human care. We anticipate that these papers will stimulate a dialogue on how to further improve care for animals in zoo and aquarium settings for topics that are either covered or not covered in this issue. Moreover, much as positive reinforcement methods developed in marine mammal facilities have recently gained more acceptance for domestic species, we hope that such species can also benefit from discussions based on the manuscripts herein.

We live in a rapidly changing world with an increasing number of wildlife extinctions. Zoos and aquariums are increasingly the sole refuges for species with little to no natural habitat, potentially serving as source populations for reintroduction into the wild or the supplementation of natural populations. The expertise that zoo and aquarium professionals have developed also serves as a unique resource for supporting conservation activities that otherwise could not occur. This expertise also compliments the work of professionals who rehabilitate wildlife in need of help due to anthropogenic or natural causes; this work has wide public support and can be viewed as the human acceptance of responsibility for anthropogenic activities that have degraded all of the world’s environments and compromised the degree to which these environments can support wildlife populations. Accordingly, many zoo and aquarium professionals have accepted the responsibility of being stewards for species conservation. In addition, these professionals have increased their focus on animal welfare and behavior as key components of contemporary (including veterinary) animal care. The inclusion of animal welfare and behavior as a critical component of animal care is a part of the complex, multidimensional considerations that zoos and aquariums face. These considerations include diverse societal values, ethical considerations, technological developments, ongoing research, improved animal care, public health considerations, disease surveillance and prevention, evolving regulations and legal concepts, economics and trade, customs, geo-politics and conflict, and environmental concerns that include climate change and habitat degradation. In response, many facilities have increased their engagement with the public to underscore the important role that zoos and aquariums play in wildlife and environmental conservation. However, the stewardship of wildlife species is complicated by local factors, economic concerns, changing priorities regarding human–animal relationships, and capacity to integrate advances in animal welfare science at both the individual and group levels.

*The path forward: why do these manuscripts matter?*

As Keulartz addressed in this Special Issue of *Animals*, there are ethical responsibilities associated with the conservation and animal care activities of zoological facilities. There can be tension between individual animal welfare and conservation needs. Zoo animal welfare concerns exist within an environment where societal perceptions of animal welfare are evolving. Increased public debate about the moral status of animals and what is best for them acknowledges that human and animal interests do not always coincide. This includes questions about whether and when animals should be under human care and the proper role of animal caretakers. However, institutions vary in the level of animal care provided and perceptions differ regarding the welfare of animals under human care. In addition, the great taxonomic diversity of animals under such care presents challenges for identifying the needs of species and refinements for individuals of a species. Therefore, there is a need for open and rigorous dialogue on how professionals can advance welfare for the animals under their care. Zoos and aquariums care for a tremendous diversity of species with a range of feeding, social, breeding, and other needs. In response to the unique and diverse needs of such animals, professional staff are increasingly trained in animal welfare and behavior in order to deliver optimal care.

*How do we get there?*

As we have suggested, zoological institutional cultures must be committed to continual improvement and critical considerations of practices. As Miller and Chinnadurai wrote, updating conceptual animal welfare models, such as going beyond the Five Freedoms model of animal welfare, is needed to make continued improvements. As they indicated, a holistic institutional approach that is *proactive* for addressing animal needs, as well as sufficiently *reactive* to address needs that may not be static, requires staff and animal training, input from multiple professionals, and ongoing assessment processes. Tallo-Parra and colleagues provided guidance for science-based welfare assessments that utilize behavioral and physiological indices of animal welfare that are supported by continued research. Moreover, Campbell-Ward echoed the need for assessments to be conducted throughout an animal’s life and the need for continued research to clarify how we assess an animal’s quality of life. These assessments are complicated by the tension that sometimes exists between the needs of individual animals and those at the population level, as discussed by DiVincenti and colleagues. These challenges extend to rehabilitation facilities that might be a part of zoo and aquarium programs or independent, where decision making is difficult regarding when animals are able to be released into the wild, need to be permanently housed under human care, or when a humane ending is required. The team of professionals led by Willette, involved in rehabilitating a range of wildlife species, developed a seminal paper on the principles of wildlife rehabilitation that are applicable to such a range. As Brando and co-authors documented, the needs of professionals that care for animals must also be addressed because without good welfare, they will struggle to meet the needs of the animals under their care.

Critically evaluating available data and distinguishing between these data and philosophical beliefs are essential for achieving optimal animal welfare. Marine mammals are a flash point for disagreements based on philosophical opposition to some or all species under human care. Jaakkola and Bruck dissected criticisms of enrichment practices for cetaceans and the merits of moving cetaceans into “sanctuaries”, respectively. They identified philosophical biases that undermine arguments in opposition to current care practices. Failure to heed rigorous assessments such as these risks will compromise animal welfare. Ironically, although not commonly considered part of public discourse about animal welfare, Henaut and Delfour point out that Sirenians are a popular group of marine mammals whose cognitive capacities do not appear to be appreciated and whose welfare concerns need further clarification. The need for critical thought extends beyond broad philosophical perspectives and must be extended to fine-scale details. One such example that is not as commonly discussed as it should be for zoo and aquarium animals is how acoustic environments affect these animals’ welfare, as addressed by Winship and Jones.

The health of animals is an important component of their welfare that must also be critically evaluated. Martelli and Krishnasamy point out that in a time where human and veterinary medicine have extraordinary diagnostic tools and therapeutic strategies at their disposal, optimal animal welfare requires thoughtfully weighing the risks, costs, and benefits of various strategies and proactively recruiting animals’ voluntary participation in their medical care. They also present the paradigm of animal wellbeing (an animal’s internal state) relative to animal welfare (the sum total of management inputs that affect wellbeing) and how recognizing these distinctions can improve the clarity of our thinking. The diagnostic tools that are used to address animal health must also be critically assessed and routinely applied where applicable, as illustrated by Hunter-Ishikawa’s bear lameness scale and Kastelic’s work on a nontraditional means of collecting blood to determine cortisol levels in ibex.

Environmental parameters are an important and basic part of animal care. There is an increasing appreciation that visitors to zoological institutions are part of the environment from the animal’s perspective and, therefore, should be considered part of welfare assessments. This point is highlighted by Bandoli’s manuscript on Asian small-clawed otters and Truax’s research on rays.

There is a need for awareness of the biases that underlie our approaches to animal welfare. Martin and colleagues discuss how the welfare of ambassador animals is often not as fully considered as the welfare of other animals and how it can be improved by reconsidering how we interact with them. The scarcity of nonmammalian papers in this Special Issue further highlights where biases can result in an incomplete consideration of many taxa’s welfare needs. Lewbart and Zachariah’s paper on invertebrates, Smith’s paper on fish, and Krönke and Xu’s paper on geckos illustrate the diversity of species for which zoological professionals must care. These papers can also help readers gain an appreciation for the need to consider what the *animals are experiencing*, rather than *what we perceive*, regardless of the challenges of evaluations based on mammals’ characteristics. The need to consider biases further extends to the cultural backgrounds of the professionals that care for animals. Ota and Yamakazi illustrate this very important point in their paper about Japanese zoos and remind us that U.S. and European-centric views of animals and their welfare may not align with other cultures’ perspectives. The Guest Co-Editors view this cultural heterogeneity as an opportunity to assess biases and view our perspectives and practices with fresh eyes. Therefore, we strived to include viewpoints from across the globe. The importance of considering the dynamic societal context where zoos and aquariums exist is emphasized by Pardo’s paper on the legal standing of animals, and how evolving case law and regulations can greatly impact whether zoos and aquariums can care for animals and how their welfare needs can be best addressed.

We thank our sponsors for their vision and leadership in supporting the invited manuscripts from leading professionals included in this Special Issue of *Animals*. Without advance knowledge of the content of the papers that were published, they committed to an open dialogue and critical consideration of how animal welfare can be further advanced. Sponsor contributions enabled all papers in this Special Issue to be Open Access and thus readily available to any reader. Their commitment to the highest standards of animal care and bettering their welfare in zoos and aquariums serves as an inspiration for the professionals that care for animals on a daily basis.

